# Association Between Pittsburgh Sleep Quality Index Scores and TIMI Frame Count-Defined Coronary Slow Flow Phenomenon in Patients with Nonobstructive Coronary Arteries: A Cross-Sectional Study

**DOI:** 10.3390/jcdd13080403

**Published:** 2026-08-21

**Authors:** Mehmet Kamil Teber, Zülfiye Kuzu, Mehmet Zafer Aydın

**Affiliations:** Department of Cardiology, Kayseri City Teaching and Education Hospital, 38080 Kayseri, Türkiye

**Keywords:** coronary slow flow, subjective sleep quality, Pittsburgh Sleep Quality Index, TIMI frame count, STOP-Bang, nonobstructive coronary arteries, coronary microvascular dysfunction

## Abstract

Coronary slow flow (CSF) is delayed distal contrast transit on angiography without flow-limiting stenosis; disturbed sleep may impair vascular control through autonomic, endothelial, inflammatory, and metabolic pathways. We evaluated Pittsburgh Sleep Quality Index (PSQI)-based sleep quality in relation to TIMI frame count (TFC)-defined CSF. This cross-sectional study enrolled 307 adults with nonobstructive coronary arteries undergoing angiography for chest pain; PSQI referenced the preceding month, and CSF was defined by corrected TFC > 27 frames in any major vessel; 132 participants had CSF and 175 normal flow. Global PSQI score was higher in CSF (median 7.0 vs. 5.0) and poor sleep quality (score > 5) was more frequent (75.8% vs. 49.7%; both *p* < 0.001). The global score correlated with mean TFC overall but not within flow groups. Each PSQI point independently raised CSF odds, including after STOP-Bang adjustment, although discrimination was modest. Sleep latency and short duration raised CSF odds; poor efficiency did not. Poorer sleep quality was independently linked to this angiographic slow-flow phenotype, mainly differentiating flow categories rather than tracking frame-count burden. Because coronary microvascular function was not measured directly, these hypothesis-generating findings should prompt systematic sleep assessment and prospective studies integrating objective sleep measures with invasive coronary physiology.

## 1. Introduction

Coronary slow flow (CSF) is recognized on coronary angiography when contrast requires an unexpectedly long time to reach distal epicardial segments in the absence of a stenosis capable of limiting flow. Tambe et al. first reported this angiographic pattern in patients with angina whose coronary arteries were otherwise normal [1]. Later studies interpreted the phenomenon as a disorder of the coronary microcirculation rather than as a benign incidental finding [2,3]. Clinical cohorts have associated CSF with recurrent chest pain and presentations resembling acute coronary syndrome [4]. Mechanistic discussions point to endothelial dysfunction, low-grade inflammation, platelet activation and vascular dysregulation, and increased resistance in the microvascular bed [5,6]. The clinical predictors reported for CSF are correspondingly cardiometabolic and hematological. In a consecutive angiographic series, male sex and higher body mass index independently predicted CSF, and high-density lipoprotein (HDL) cholesterol was lower in affected patients [4]. A recent meta-analysis of 23 studies, comprising 2309 patients with CSF and 3377 controls, identified current smoking, higher body mass index, higher triglyceride and total cholesterol concentrations, higher platelet and white blood cell counts, and a higher platelet-to-lymphocyte ratio as risk factors, and framed these as potentially modifiable targets [7]. Sleep quality has not been examined among these predictors. The Thrombolysis in Myocardial Infarction (TIMI) frame count (TFC) provides a reproducible way to quantify flow by enumerating cine frames until contrast reaches predefined distal landmarks; the longer left anterior descending artery is conventionally adjusted by dividing the raw count by 1.7 [8].

Sleep is increasingly viewed as a core component of cardiovascular health, not simply a lifestyle background variable. The Pittsburgh Sleep Quality Index (PSQI) is a commonly used self-report tool that captures perceived sleep quality and sleep-related problems during the previous month [9], and its Turkish form has been validated for clinical and research use [10]. The total score extends from 0 to 21; higher scores reflect worse sleep, and scores above 5 are often used to classify poor sleep quality [9]. Current cardiovascular health frameworks explicitly include sleep duration, while adult consensus recommendations generally identify 7 h per night as a minimum target for healthy sleep [11,12].

Several converging pathways make a sleep-CSF relationship plausible. Short, delayed, fragmented, or otherwise disturbed sleep may enhance sympathetic drive, reduce nitric oxide-dependent endothelial vasodilation, and increase oxidative and inflammatory activity [13]. Sleep research in cardiovascular populations also connects poor sleep with platelet behavior and unfavorable metabolic regulation [14]. These mechanisms overlap with leading explanations for CSF, particularly heightened microvascular tone and diffuse abnormalities in vasomotor regulation [5,6]. Predictive-factor studies further support shared inflammatory and metabolic vulnerability [7].

Evidence directly addressing multidimensional sleep quality in CSF remains sparse. Existing CSF studies have more often examined obstructive sleep apnea or sleep-disordered breathing risk [15,16]. Other cross-sectional work has related CSF to psychological factors such as anxiety and depression [17,18], and a newer report added that adverse childhood experiences and alexithymia were not associated with CSF [19]. In wider coronary populations, poor PSQI-assessed sleep or short sleep has been linked to incident coronary heart disease [20], angiographic lesion severity and prognosis in young adults with acute coronary syndrome [21], progression of chronic coronary artery disease [22], and lower coronary microvascular function [23]. Together, these data suggest a coronary-sleep connection, but they do not establish whether subjective sleep quality differentiates TFC-defined CSF from normal flow among patients without obstructive epicardial disease.

The primary hypothesis was that a higher global PSQI score, denoting poorer subjective sleep quality, would be independently associated with increased odds of TFC-defined CSF after adjustment for demographic and cardiometabolic covariates; the corresponding null hypothesis was that no independent association exists. The secondary hypothesis was that, if this relationship were dimensional, the global PSQI score would also correlate with vessel-specific and mean TFC within each predefined flow group. No directional hypothesis was specified for the individual PSQI components, which were examined in exploratory analyses. The main aim was to test whether a higher global PSQI score was independently related to TFC-defined CSF. A second aim was to determine whether the association represented a graded relationship with frame counts or primarily a difference between predefined angiographic flow phenotypes. Exploratory component analyses were then used to identify sleep domains most strongly aligned with CSF.

## 2. Materials and Methods

### 2.1. Ethics Approval and Consent to Participate

The protocol was approved by the Ethics Committee of Kayseri City Teaching and Research Hospital (approval number: 706; approval date: 16 December 2025). All participants provided written informed consent before entering the study. The investigation followed the Declaration of Helsinki and relevant national regulations, and no patient identifiers were included in analytic outputs or in the manuscript.

### 2.2. Study Design and Population

This single-center cross-sectional observational study was carried out in the Department of Cardiology at Kayseri City Hospital. Consecutive adults who presented with chest pain and underwent diagnostic coronary angiography from December 2025 through May 2026 were screened. Nonobstructive coronary arteries were defined by <50% luminal narrowing in every major epicardial vessel. Individuals younger than 18 years were not eligible.

All angiographic procedures were elective and were performed for suspected chronic coronary syndrome. Invasive angiography was not undertaken on the basis of symptoms alone: the clinical indication in every case was an abnormal exercise electrocardiographic stress test, an abnormal myocardial perfusion scintigraphy result, or both, in a patient who had presented with chest pain. Patients presenting with an acute coronary syndrome, as well as those with an acute coronary syndrome within the preceding 3 months, previous myocardial infarction, or previous coronary revascularization, were not eligible (Section 2.3). The analytic population therefore consisted exclusively of stable, symptomatic patients undergoing a first invasive diagnostic evaluation after an abnormal noninvasive ischemia test. Because this indication applied uniformly across the cohort, and because obstructive epicardial disease was excluded in all participants by the requirement for <50% luminal narrowing in every major vessel, the CSF and normal-flow groups were drawn from an identical referral pathway and did not differ with respect to the category of indication. The proportion of patients referred on the basis of exercise electrocardiography as opposed to myocardial perfusion scintigraphy was not separately recorded in the analytic dataset, and this more granular breakdown could therefore not be compared between flow groups.

Eligible participants were consecutive adults (aged ≥ 18 years) who presented with chest pain, underwent elective diagnostic coronary angiography for suspected chronic coronary syndrome after an abnormal noninvasive ischemia test, and were found to have nonobstructive coronary arteries, defined by <50% luminal narrowing in every major epicardial vessel. Patients were excluded if they had conditions that could confound the assessment of coronary flow or of sleep, or that indicated an acute or structural cardiac disorder, including recent or previous ischemic events and coronary procedures, structural heart disease, systemic inflammatory, hematologic, malignant, renal, or hepatic conditions, and psychiatric, cognitive, or sleep-related comorbidity; the specific criteria are listed in Section 2.3.

### 2.3. Exclusion Criteria

Exclusion criteria were selected to limit confounding related to recent ischemic episodes, prior coronary procedures, structural cardiac disease, systemic inflammation, hematologic conditions, malignancy, advanced renal or hepatic impairment, and psychiatric, cognitive, or sleep-related comorbidity. Patients were ineligible if they had acute coronary syndrome within the preceding 3 months, previous myocardial infarction, prior percutaneous coronary intervention or coronary artery bypass grafting, dilated, hypertrophic, or restrictive cardiomyopathy, moderate-to-severe valvular disease, decompensated heart failure, coronary spasm, coronary aneurysm, or coronary dissection. Further exclusions were active or chronic infection, autoimmune or chronic inflammatory disease, clinically relevant anemia, hematologic disease, malignancy, advanced kidney or liver dysfunction, previously documented obstructive sleep apnea, a previously diagnosed sleep disorder other than obstructive sleep apnea requiring ongoing evaluation or treatment, and a psychiatric or cognitive disorder.

### 2.4. Coronary Angiography and Tfc Evaluation

Coronary angiography was performed via radial or femoral access as part of routine care. Cineangiographic acquisitions were recorded at 30 frames/s and evaluated offline. TFC was determined according to the quantitative approach reported by Gibson et al. [8]. Counting started at the frame in which contrast first fully entered the proximal artery and stopped when contrast reached a prespecified distal landmark. The distal landmarks were the terminal bifurcation, also described as the pitchfork, in the left anterior descending artery; the distal obtuse marginal branch in the left circumflex artery; and the first posterolateral branch in the right coronary artery.

Epicardial flow was first graded visually using the TIMI flow grade classification. All 307 participants had TIMI grade 3 flow in every major epicardial vessel, and no participant had TIMI grade 0, 1, or 2 flow; this is consistent with the exclusion of obstructive stenosis, coronary thrombus, dissection, and coronary spasm, since TIMI grades below 3 are operationally defined with reference to an obstruction. Because the TIMI flow grading system can vary between and within observers, and because it cannot discriminate among patients who all have grade 3 flow, the corrected TIMI frame count (TFC) was also calculated for each patient. For the left anterior descending artery (LAD), the raw frame count was divided by 1.7 to obtain the corrected count [8]; counts from the left circumflex artery (LCx) and right coronary artery (RCA) were left unadjusted. Vessel-level measures are presented as corrected LAD TFC, LCx TFC, and RCA TFC. TFC is an angiographic index of epicardial contrast transit and does not itself measure coronary microvascular function; no direct assessment of microvascular physiology, such as coronary flow reserve, the index of microcirculatory resistance, or acetylcholine provocation, and no intracoronary imaging were performed. Accordingly, the outcome of this study is an angiographically defined coronary slow-flow phenotype rather than invasively confirmed coronary microvascular dysfunction. For descriptive summaries and correlation analyses, mean TFC was derived from the average of the three vessel values; however, mean TFC alone was not used to assign flow category. CSF was defined when any corrected LAD, LCx, or RCA TFC was >27 frames in a patient without obstructive stenosis. Normal coronary flow required corrected LAD TFC ≤ 27 frames, LCx TFC ≤ 27 frames, and RCA TFC ≤ 27 frames.

### 2.5. Sleep Quality Assessment

Subjective sleep quality was measured with the PSQI, a self-completed questionnaire evaluating sleep during the previous month [9]. The validated Turkish version was administered [10]. Participants filled in the questionnaire after coronary angiography and before hospital discharge, and they were specifically instructed to answer with reference to the month preceding the procedure. This timing was considered during interpretation, because peri-procedural circumstances may have affected recall of sleep symptoms.

The PSQI global score can vary from 0 to 21, with higher values denoting poorer sleep. A global score >5 was used for descriptive classification of poor sleep quality [9]. The instrument includes seven component scores: subjective sleep quality, sleep latency, sleep duration, habitual sleep efficiency, sleep disturbances, use of sleep medication, and daytime dysfunction. Each component is scored from 0 to 3. Prespecified binary sleep-domain indicators were latency >30 min, sleep duration < 6 h, sleep duration < 7 h, and sleep efficiency < 85%. The <7 h threshold was retained because 7 h is widely regarded as the lower boundary for recommended adult sleep duration [11,12].

### 2.6. Stop-Bang Assessment

The STOP-Bang total score was derived as a questionnaire-based estimate of obstructive sleep apnea risk; because the STOP questionnaire was originally designed for obstructive sleep apnea screening, higher scores indicate a greater likelihood of obstructive sleep apnea, and a systematic review with meta-analysis has supported its screening performance across different geographic settings [24,25,26]. The eight yes/no items assess snoring, tiredness during the day, observed apnea, high blood pressure or antihypertensive treatment, body mass index > 35 kg/m^2^, age > 50 years, neck circumference > 40 cm, and male sex. Each affirmative response adds 1 point, so the total score ranges from 0 to 8. A score ≥ 3 was summarized descriptively as an elevated screening-risk category.

Although participants with previously documented obstructive sleep apnea were not enrolled (Section 2.3), STOP-Bang was retained to capture residual, undiagnosed obstructive sleep apnea screening risk. This approach was clinically relevant because obstructive sleep apnea is associated with cardiovascular disease and shares autonomic, endothelial, inflammatory, and metabolic pathways with coronary vascular dysfunction [27]. STOP-Bang was included mainly in sensitivity analyses rather than in the primary model, because several of its components duplicate model covariates such as age, sex, body mass index, hypertension, and neck circumference. These sensitivity analyses evaluated whether residual obstructive sleep apnea screening risk, as captured by a practical bedside tool, changed the relationship between global PSQI score and CSF.

### 2.7. Clinical and Laboratory Variables

Demographic data, cardiovascular risk factors, laboratory results, and available medication variables were obtained from the analytic dataset. Body mass index was analyzed in kg/m^2^. The prespecified primary clinical model contained global PSQI score, age, sex, body mass index, current smoking, diabetes mellitus, hypertension, and hyperlipidemia. Triglycerides and HDL cholesterol were assessed in a sensitivity model. Medication variables were described separately and entered into sensitivity analyses rather than the primary model. Medication variables comprised angiotensin-converting enzyme (ACE) inhibitor or angiotensin receptor blocker (ARB) use, beta blocker use, calcium channel blocker use, diuretic use, statin use, aspirin use, additional antiplatelet use, oral anticoagulant use, and antidiabetic therapy.

### 2.8. Statistical Analysis

Statistical analyses were performed using SPSS version 27.0 (IBM Corp., Armonk, NY, USA). Analytic code is available from the corresponding author upon reasonable request. This study was not registered in a public trial registry. Continuous variables were first examined for distributional patterns. Because PSQI scores, TFC values, STOP-Bang score, and several laboratory measures showed non-normal distributions, continuous data are summarized as median [interquartile range (IQR)] and compared with Mann–Whitney U tests. Categorical data are shown as *n*/*N* (%) and compared with Pearson chi-square tests or, when small expected cell counts were present, Fisher exact tests.

Spearman methods were used to evaluate associations between global PSQI score and vessel-specific as well as mean TFC. Because an apparent pooled correlation may simply reflect separation between predefined flow groups, the same correlations were examined separately in the CSF and normal-flow groups. Flow group-adjusted partial Spearman correlations were obtained by correlating rank residuals after accounting for flow group membership.

The primary relationship between global PSQI score and CSF was modeled with multivariable logistic regression. Given the consecutive observational design, no a priori sample size calculation was performed; the achieved sample size was determined by eligible enrollment during the study period rather than by a predetermined target. To document the adequacy of the achieved sample for the primary hypothesis, sample size and sensitivity analyses were subsequently performed with G*Power 3.1.9.4 (Heinrich Heine University, Düsseldorf, Germany) using the z test for logistic regression with a normally distributed covariate, applying the large-sample approximation with variance correction [28,29]. Input parameters were a two-sided α of 0.05, 80% power, an outcome probability of 0.43 under the null hypothesis, and a squared multiple correlation of 0.066 between global PSQI score and the remaining covariates of the primary model. Under these assumptions, detection of the observed association (odds ratio 2.22 per 1 standard deviation [SD] increase in global PSQI score, equivalent to 1.58 per 1-point increase) would have required 89 participants, whereas detection of a more conservative effect of 1.58 per 1-SD increase would have required 177 participants; the achieved sample of 307 participants exceeded both requirements. In the corresponding sensitivity analysis, 307 participants provided 80% power to detect an odds ratio as small as 1.41 per 1-SD increase in global PSQI score, equivalent to 1.22 per 1-point increase, which is well below the observed effect. The primary model also satisfied conventional criteria for model stability, with 132 outcome events and 8 covariates, corresponding to 16.5 events per variable [30]. Estimates are presented as odds ratios with 95% confidence intervals. Model evaluation included variance inflation factors, the Hosmer–Lemeshow goodness-of-fit test, apparent area under the receiver operating characteristic (ROC) curve (AUC), and bootstrap confidence intervals for discrimination. A ROC analysis also examined how well global PSQI score alone separated CSF from normal coronary flow. The threshold selected by the Youden index was considered an internally derived exploratory value rather than a validated diagnostic cutoff.

Analyses of PSQI components were exploratory. For binary sleep-domain variables and ordinal component scores, separate adjusted logistic regression models were built with CSF as the outcome. These models used the same clinical covariates as the primary model but omitted the global score to prevent collinearity. Multiple testing was handled with the Benjamini–Hochberg false discovery rate (FDR) method [31]. All 307 participants had complete data for the primary model, so imputation was not required. For variables with item-specific missingness, complete-case analysis was used and denominators were reported. Reporting followed the Strengthening the Reporting of Observational Studies in Epidemiology (STROBE) guidance [32]. Statistical significance was assessed with two-sided *p* values < 0.05.

## 3. Results

### 3.1. Study Cohort and Baseline Profile

**Figure 1:**
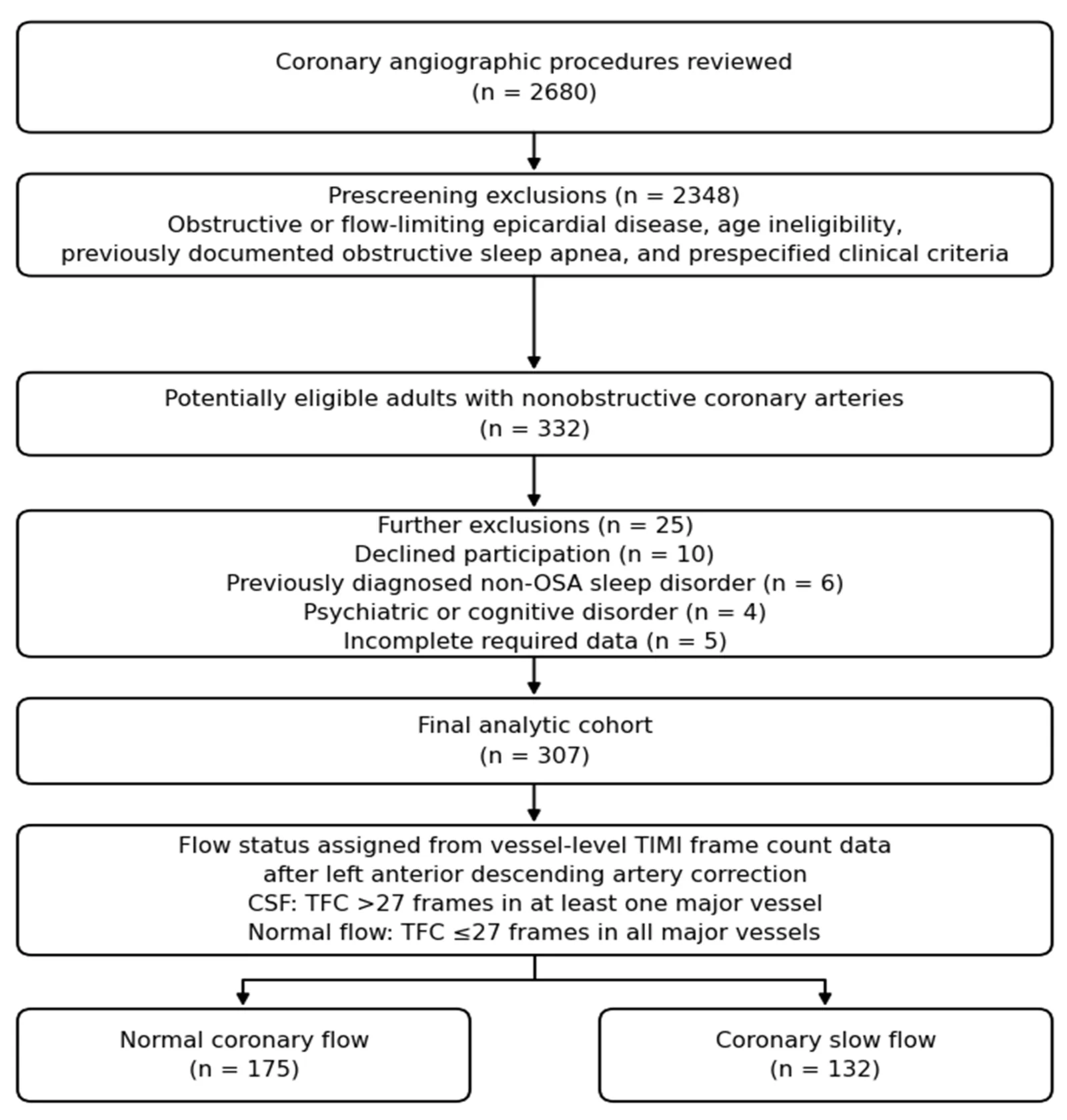
Selection of patients and analytic cohort flow. Previously documented obstructive sleep apnea was addressed during prescreening. Final flow category was determined from vessel-level TFC using corrected LAD TFC, LCx TFC, and RCA TFC values. CSF was defined by TFC > 27 frames in any vessel, whereas normal flow required corrected LAD TFC ≤ 27 frames, LCx TFC ≤ 27 frames, and RCA TFC ≤ 27 frames. CSF, coronary slow flow; LAD, left anterior descending artery; LCx, left circumflex artery; OSA, obstructive sleep apnea; RCA, right coronary artery; TFC, TIMI frame count.

Across the enrollment interval, 2680 angiographic procedures were reviewed. Prescreening with angiographic, age-related, and predefined clinical criteria, including previous documentation of obstructive sleep apnea, identified 332 adults with nonobstructive coronary arteries who were potentially eligible. Another 25 patients were removed from consideration because they declined participation, had a previously diagnosed sleep disorder other than obstructive sleep apnea requiring ongoing evaluation or treatment, had a psychiatric or cognitive disorder, or had missing required data. The final analytic sample consisted of 307 participants. Classification based on vessel-level TFC values after LAD correction identified 132 patients with CSF and 175 with normal coronary flow (Figure 1). Across the cohort, age ranged from 25 to 89 years, and global PSQI scores ranged from 3 to 11.

Table 1 presents baseline characteristics. Age and body mass index were similar across the two flow groups. Male sex and current smoking were more common among patients with CSF. Fasting glucose, triglycerides, hemoglobin, monocyte count, and platelet distribution width were higher in the CSF group, whereas HDL cholesterol and plateletcrit were lower. Albumin, diabetes mellitus, hypertension, hyperlipidemia, chronic obstructive pulmonary disease, estimated glomerular filtration rate, low-density lipoprotein (LDL) cholesterol, C-reactive protein (CRP), glycated hemoglobin (HbA1c), neutrophil count, platelet count, lymphocyte count, red cell distribution width, and mean platelet volume were not significantly different between groups.

Epicardial TIMI flow grade was 3 in every major vessel in all 307 participants, in both the CSF and the normal-flow group; no participant had a TIMI flow grade below 3. Consistent with the definition of flow status, vessel-specific and mean TFC values were greater in the CSF group. Median mean TFC was 29.0 [26.9 to 32.1] among patients with CSF and 20.7 [19.5 to 21.7] among those with normal coronary flow (*p* < 0.001). Within the CSF group, one-vessel involvement was present in 67 patients (50.8%), two-vessel involvement in 41 (31.1%), and three-vessel involvement in 24 (18.2%).

### 3.2. Primary Psqi Findings

**Figure 2:**
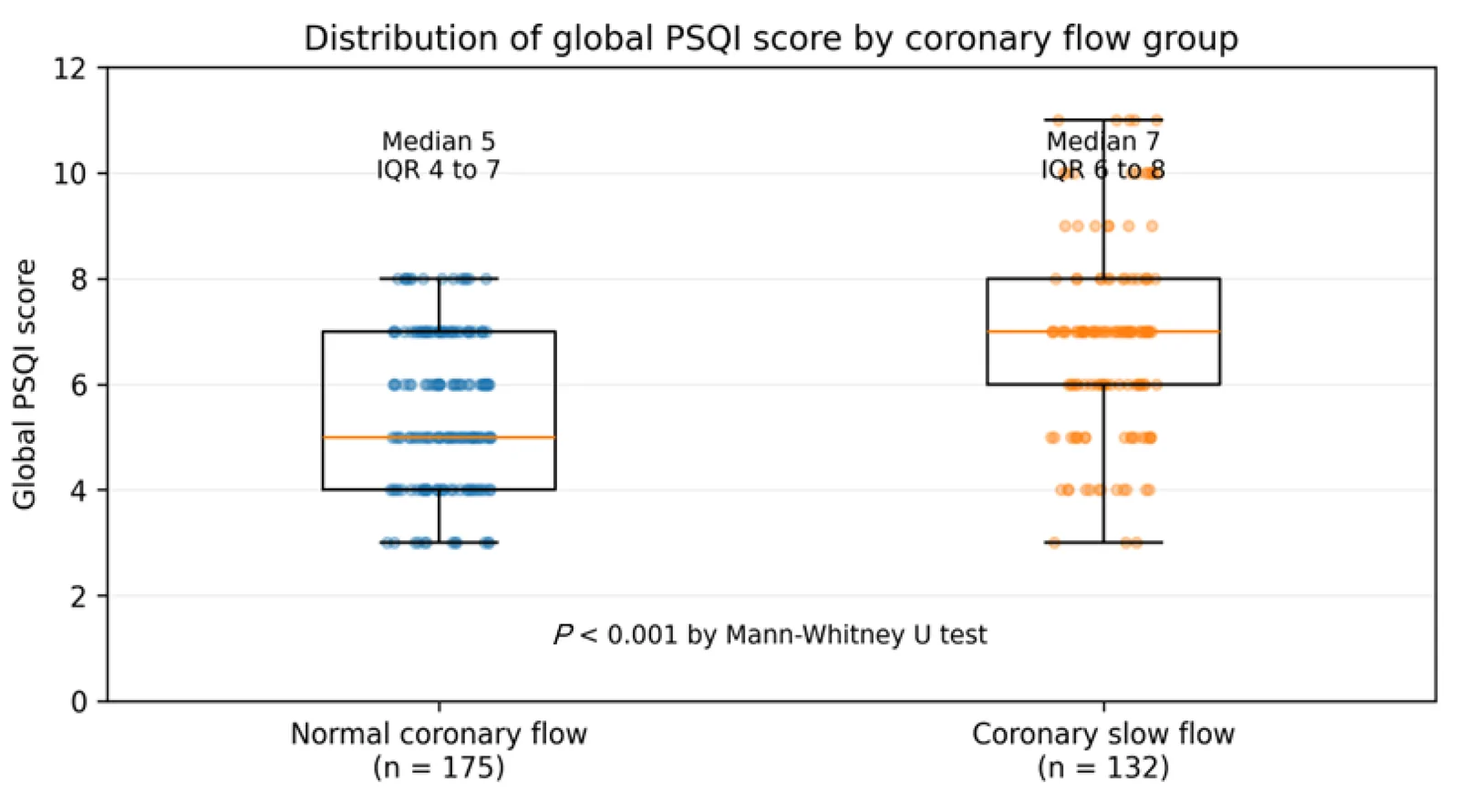
Global PSQI score distribution by TFC-defined flow category. Box plots display medians(orange line) and interquartile ranges, with individual observations overlaid. By Mann–Whitney U testing, global scores were higher in the CSF group than in the normal-flow group. PSQI, Pittsburgh Sleep Quality Index.

Sleep quality varied according to angiographic flow phenotype. The median global PSQI score was 7.0 [6.0 to 8.0] in patients with CSF and 5.0 [4.0 to 7.0] in patients with normal coronary flow (*p* < 0.001; Figure 2). Poor sleep quality, defined by global score > 5, was observed in 100 of 132 patients with CSF (75.8%) compared with 87 of 175 patients with normal flow (49.7%; *p* < 0.001).

**Figure 3:**
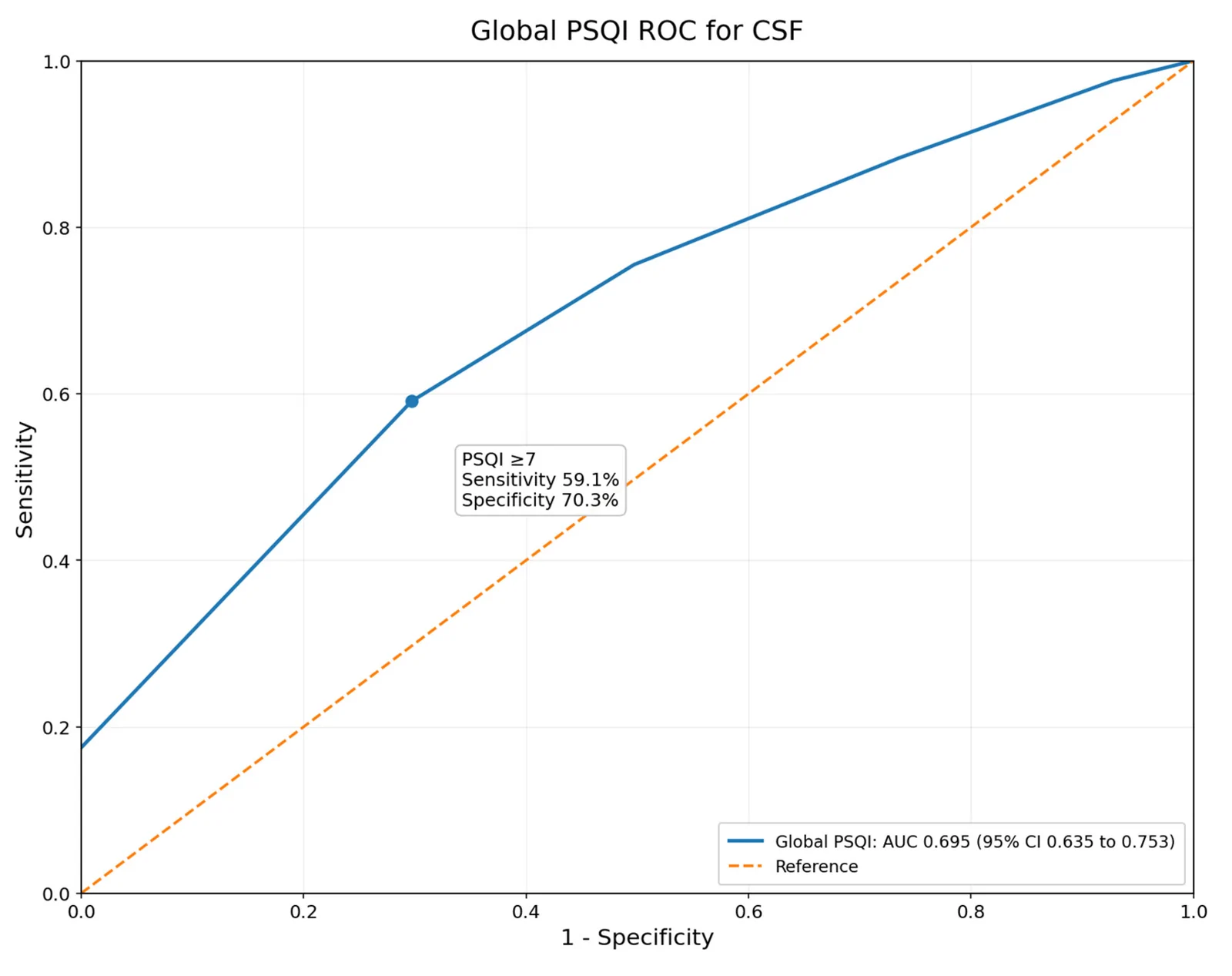
Exploratory receiver operating characteristic analysis of global PSQI score for differentiating CSF from normal coronary flow. The AUC was 0.695 (bootstrap 95% CI 0.635 to 0.753). The study-derived Youden threshold was PSQI ≥ 7, yielding 59.1% sensitivity and 70.3% specificity; this threshold requires external validation. AUC, area under the curve; CI, confidence interval; PSQI, Pittsburgh Sleep Quality Index.

When considered by itself, the global PSQI score had modest ability to discriminate CSF from normal flow, with an area under the receiver operating characteristic curve of 0.695 (bootstrap 95% confidence interval 0.635 to 0.753; Figure 3). The internally selected Youden threshold was PSQI ≥ 7, corresponding to 59.1% sensitivity, 70.3% specificity, 60.0% positive predictive value, and 69.5% negative predictive value. Because this threshold was generated from the study dataset, external validation is needed before clinical use.

### 3.3. Correlations Between Psqi and Tfc

Table 2 summarizes the Spearman correlation analyses. In the full cohort, the global PSQI score was positively correlated with corrected LAD TFC, LCx TFC, RCA TFC, and mean TFC. For mean TFC, the pooled correlation coefficient was rho = 0.292 (*p* < 0.001).

After stratification by flow status, mean TFC no longer correlated with the global score in the CSF group (rho = −0.075, *p* = 0.393) or the normal-flow group (rho = 0.111, *p* = 0.145). The association was also absent after adjustment for flow group membership (partial rho = 0.013, *p* = 0.818). Vessel-specific subgroup correlations that reached nominal significance were small, inconsistent in direction, and not preserved after flow group adjustment.

The anatomical extent of slow flow was examined in the same way, by comparing global PSQI score across the number of vessels with a corrected TFC >27 (Supplementary Table S8). Across the whole cohort the score differed between categories (Kruskal–Wallis *p* < 0.001), but this reflected the separation between patients with no affected vessel and those with any affected vessel. Within the CSF group, global PSQI score did not rise with the number of affected vessels: median scores were 7.0 [6.0 to 8.0] for one-vessel involvement, 7.0 [6.0 to 8.0] for two-vessel involvement, and 5.5 [4.0 to 7.0] for three-vessel involvement (Kruskal–Wallis *p* = 0.082; Spearman rho = −0.134, *p* = 0.125). This pattern parallels the frame-count correlations and again indicates that subjective sleep quality distinguishes the CSF phenotype from normal coronary flow rather than grading its angiographic extent.

### 3.4. Multivariable and Sensitivity Analyses

In the primary clinical logistic regression model, which included all 307 participants, the global PSQI score remained independently related to CSF after adjustment for age, sex, body mass index, current smoking, diabetes mellitus, hypertension, and hyperlipidemia (Table 3). Each additional point in global score was associated with a 58% increase in the odds of CSF (adjusted odds ratio 1.58, 95% confidence interval 1.34 to 1.88, *p* < 0.001). Current smoking, male sex, and age also showed independent associations with CSF, whereas body mass index, diabetes mellitus, hypertension, and hyperlipidemia did not.

The primary model did not show problematic multicollinearity; all variance inflation factors were <1.3. Apparent discrimination was moderate, with an area under the curve of 0.760 (bootstrap 95% confidence interval 0.722 to 0.825). The Hosmer–Lemeshow statistic indicated imperfect calibration (chi-square 20.40, *p* = 0.009). Model findings were interpreted primarily as associations.

Because the indication for angiography was identical for every participant, it was invariant across the cohort and could not be entered as a covariate. To examine whether the independent determinants of CSF were altered by more complete adjustment, an extended model was therefore fitted in which every baseline variable that differed between flow groups in Table 1 was added to the primary covariate set (Supplementary Table S7). Global PSQI score remained independently associated with CSF in this model (adjusted odds ratio 1.59 per point, 95% confidence interval 1.33 to 1.90, *p* < 0.001), as did age, current smoking, triglycerides, HDL cholesterol, monocyte count, and plateletcrit; apparent discrimination increased to an area under the curve of 0.829 and calibration was adequate (Hosmer–Lemeshow *p* = 0.697). Because this model contained 15 covariates for 132 events, corresponding to 8.8 events per variable, it is reported as an exploratory sensitivity analysis rather than as a replacement for the primary model.

Supplementary Table S3 reports the sensitivity analyses. The global PSQI estimate remained robust when STOP-Bang total score was added, when the STOP-Bang ≥ 3 category was added, when body mass index or hyperlipidemia was removed, when triglycerides and HDL cholesterol were included, and when statin and oral anticoagulant variables were entered. STOP-Bang total score and STOP-Bang ≥ 3 screening category did not differ significantly between groups (Supplementary Table S5). A binary model using poor sleep quality also supported the primary result: after clinical adjustment, global score > 5 was associated with CSF (adjusted odds ratio 3.08, 95% confidence interval 1.81 to 5.25, *p* < 0.001).

Pharmacotherapy in use before coronary angiography was examined in relation to both flow category and frame count (Supplementary Table S9). Of the nine medication classes recorded, only oral anticoagulant use differed between groups, being more frequent among patients with CSF (13.6% versus 5.7%, *p* = 0.017); this difference was attenuated to borderline significance after clinical adjustment (adjusted odds ratio 2.52, 95% confidence interval 1.00 to 6.39, *p* = 0.051). Oral anticoagulant users also had a higher mean TFC than non-users (26.7 [21.9 to 30.6] versus 22.0 [20.3 to 27.8] frames, *p* = 0.007). Aspirin use showed a borderline inverse association with CSF after adjustment (adjusted odds ratio 0.57, 95% confidence interval 0.33 to 1.00, *p* = 0.050), although the crude prevalence did not differ between groups and mean TFC did not differ by aspirin use. Renin-angiotensin system inhibitors, beta blockers, calcium channel blockers, diuretics, statins, additional antiplatelet agents, and antidiabetic therapy were unrelated to flow category and to mean TFC. Antidiabetic therapy was almost perfectly collinear with the diabetes covariate, differing in only 1 of 307 patients, and was therefore modelled in place of that covariate rather than alongside it. Importantly, when all medication variables were entered into the primary model simultaneously, the global PSQI estimate was unchanged (adjusted odds ratio 1.57, 95% confidence interval 1.32 to 1.87, *p* < 0.001).

### 3.5. Psqi Component Analyses

Supplementary Table S2 displays component distributions, and Table 4 gives the exploratory adjusted component models. After clinical adjustment and false discovery rate correction, the sleep-domain variables associated with CSF were sleep latency > 30 min, sleep duration < 6 h, sleep duration < 7 h, ordinal sleep latency score, and ordinal sleep duration score. Sleep efficiency, subjective sleep quality, sleep disturbances, sleep medication use, and daytime dysfunction were not statistically significant after adjustment and false discovery rate correction. Supplementary Table S4 shows selected domain correlations with mean TFC; similar to the global score, pooled associations were not sustained after stratification or adjustment for flow group.

## 4. Discussion

This cross-sectional single-center analysis showed that poorer sleep quality measured by the PSQI was independently associated with TFC-defined CSF in adults whose coronary arteries were nonobstructive. The association was observed for the continuous global score and for the conventional poor-sleep category, persisted after adjustment for key demographic and cardiometabolic factors, and was essentially unchanged in analyses incorporating STOP-Bang. Importantly, the results were obtained in a symptomatic angiography population, so the inference relates to patients undergoing clinical evaluation rather than to community screening. Equally important, the outcome was an angiographically defined slow-flow phenotype based on TFC, and coronary microvascular function was not assessed by coronary flow reserve, the index of microcirculatory resistance, acetylcholine provocation, or intracoronary imaging. The present findings therefore describe an association between sleep quality and an angiographic phenotype, not a demonstrated association between sleep quality and coronary microvascular dysfunction, and should be regarded as hypothesis-generating throughout.

The pattern of correlations helps frame the finding. Although the global score correlated with mean TFC in the pooled sample, correlations with mean frame count were no longer present after patients were examined within flow-defined groups or after flow group membership was accounted for. Some vessel-level subgroup correlations reached nominal significance, but the magnitudes were small and the directions were not consistent. Thus, the most cautious interpretation is that PSQI score differentiates the CSF phenotype from normal coronary flow, rather than quantifying angiographic severity within either phenotype. This distinction is important because a variable that separates groups may not necessarily track the biological intensity of the same condition once group membership has already been assigned.

Our findings extend this literature rather than repeating it. Instead of re-examining the cardiometabolic and hematological predictors already reported for CSF [5,6,7,33,34,35], or the obstructive sleep apnea focus of previous sleep studies in this setting [15,16], the present work uses multidomain subjective sleep quality as the principal exposure in a cohort defined by TFC. Mechanistically, poor or short sleep is thought to raise sympathetic outflow and circulating catecholamines, attenuate nitric oxide-dependent endothelial vasodilation, and promote oxidative stress, low-grade inflammation, and platelet activation [13,14]. Each of these processes can increase resting microvascular tone and coronary microvascular resistance, which is the level at which CSF is thought to originate; delayed contrast transit through angiographically smooth epicardial arteries would then represent the visible expression of diffuse distal vasomotor dysfunction rather than a discrete epicardial obstruction [5,6]. This mechanistic chain also offers a coherent explanation for the domains most strongly associated with CSF in our data, since prolonged sleep latency and short sleep duration are the features most closely linked to sustained nocturnal sympathetic activation. This account remains inferential, however: because we characterised an angiographic slow-flow phenotype and did not measure microvascular function directly, the proposed pathways are hypothesised rather than demonstrated, and the mechanistic interpretation should be read as tentative. Notably, although systemic inflammation is a proposed link, C-reactive protein did not differ between flow groups, which may reflect its limited sensitivity to localized microvascular inflammation, insufficient statistical power, or a genuinely limited role. The design cannot establish directionality: poor sleep could contribute to vascular dysregulation, follow recurrent chest pain and related distress, or reflect shared upstream factors.

Pre-procedural pharmacotherapy did not account for the findings. No class of cardiovascular or antidiabetic medication was independently related to flow category, and the association between global PSQI score and CSF was unchanged when all medication variables were included simultaneously. Oral anticoagulant use was the only exception, being more frequent in the CSF group and associated with a higher mean TFC. We interpret this cautiously as confounding by indication rather than as a pharmacological effect on epicardial flow, because oral anticoagulants are prescribed for atrial fibrillation and venous thromboembolism rather than for coronary disease, because the association was only borderline after adjustment, and because the number of treated patients was small. Data on the underlying anticoagulation indication and on rhythm status were not available, so this observation should be regarded as hypothesis-generating.

Adding STOP-Bang total score did not weaken the association between global PSQI score and CSF. This suggests that, in this dataset, the relationship was not simply explained by questionnaire-based obstructive sleep apnea risk. Even so, STOP-Bang is a screening tool and cannot substitute for polysomnography or home sleep apnea testing [24,25,26]. Undetected sleep-disordered breathing may still be present in some patients with CSF, especially because obstructive sleep apnea has strong cardiovascular associations and shares mechanisms relevant to coronary microvascular dysfunction [27].

The component-level analyses provide additional clinical context. Sleep latency and sleep duration were the domains most consistently related to CSF, whereas sleep efficiency, sleep medication use, and daytime dysfunction were not independently associated after adjustment. This pattern indicates that difficulty falling asleep and insufficient sleep time may be particularly relevant to the CSF phenotype in this cohort. Because sleep disturbances did not remain significant after false discovery rate correction, the domain-level results should be viewed as exploratory and should not replace interpretation of the global PSQI score.

From a clinical perspective, the PSQI should not be treated as a diagnostic test for CSF. Its more appropriate role is to support a structured sleep history in symptomatic patients with nonobstructive coronary arteries. Such an assessment can address insomnia symptoms, total sleep time, sleep timing, snoring, witnessed apneas, daytime sleepiness, shift work, caregiving demands, caffeine and alcohol exposure, sedative or hypnotic medications, pain, depression, and anxiety. The questionnaire may therefore serve as a practical prompt for discussing modifiable sleep problems, while angiographic and physiologic evaluation remain necessary for coronary diagnoses. When chronic insomnia is suspected, evidence-based behavioral therapy such as cognitive behavioral therapy for insomnia may be considered [36]. If obstructive sleep apnea is suspected and verified with objective testing, management should follow guideline-based care [27].

Several methodological features should be noted. Flow status was determined from vessel-level TFC values after LAD correction using a predefined >27-frame criterion. Sleep was assessed with a validated multidimensional questionnaire. The analysis incorporated nonparametric summaries, multivariable adjustment, model diagnostics, sensitivity models, receiver operating characteristic analysis, and false discovery rate correction for component testing. By separately examining pooled, within-group, and flow-adjusted correlations, the analysis reduced the risk of misinterpreting a pooled frame-count association as evidence of severity tracking within already defined flow phenotypes. The same analytic framework also allowed the global score and individual sleep domains to be interpreted without overstating exploratory component findings.

Several limitations merit emphasis. Because this was a cross-sectional study from a single center, generalizability is limited and temporal or causal conclusions cannot be made. The primary model also showed imperfect calibration (Hosmer–Lemeshow *p* = 0.009), indicating that predicted probabilities may not align precisely with observed outcomes across all risk strata; findings should therefore be interpreted as evidence of association rather than as a validated prediction tool. The comparator group consisted of symptomatic patients referred for angiography rather than community controls. Although the indication for angiography was uniform across the cohort, namely an abnormal exercise electrocardiographic stress test and/or an abnormal myocardial perfusion scintigraphy result in a patient with chest pain, the relative contribution of each noninvasive modality and the formal grading of anginal symptoms were not recorded at the individual patient level and could not be compared between the CSF and normal-flow groups. Residual differences in the intensity or character of presenting symptoms between the two groups therefore cannot be excluded, and prospective studies should record standardized symptom and indication data. The PSQI was completed after angiography while referring to sleep over the previous month; therefore, responses may have been influenced by procedural context, symptom intensity, or anxiety. Although previously documented obstructive sleep apnea was excluded, objective sleep testing was not performed, so unrecognized sleep-disordered breathing remains possible. Residual confounding may also have arisen from unmeasured factors such as mood symptoms, shift work, caffeine or alcohol use, sedative exposure, pain, physical activity, and socioeconomic stress. Because sleep quality was self-reported, measurement error and reporting bias cannot be excluded despite use of a validated instrument.

Angiographic evaluation relied on final consensus TFC values obtained by two blinded interventional cardiologists using predefined landmarks. Formal interobserver reliability statistics and detailed procedural variables, including vasodilator administration, hemodynamics, contrast injection characteristics, and acquisition projections, were not available. The most important conceptual limitation concerns the nature of the outcome. CSF was defined exclusively by the angiographic TIMI frame count, without any direct assessment of coronary microvascular function, such as coronary flow reserve, the index of microcirculatory resistance, or acetylcholine provocation, and without intracoronary imaging to characterise the underlying coronary substrate. The study therefore cannot demonstrate an association between sleep quality and coronary microvascular dysfunction itself, but only between sleep quality and an angiographic slow-flow phenotype. This distinction is not merely terminological: recent evidence indicates that angiographic slow flow is not a valid surrogate for invasively diagnosed coronary microvascular dysfunction in all patients [37]. The present findings should accordingly be interpreted as hypothesis-generating, and the mechanistic interpretation offered above should be regarded as provisional. They provide the rationale for future multicentre prospective studies that integrate standardized objective sleep assessment, using actigraphy, home sleep apnea testing, or polysomnography, with invasive coronary physiology and, where appropriate, intracoronary imaging. Repeated sleep and vascular measurements would also help determine whether changes in sleep status occur before, after, or in parallel with changes in coronary flow behavior. Longitudinal and interventional designs are necessary to determine whether improving sleep can modify symptoms, coronary flow indices, or coronary microvascular physiology.

## 5. Conclusions

In adults with nonobstructive coronary arteries, poorer PSQI-assessed subjective sleep quality was independently associated with a TFC-defined, angiographically diagnosed coronary slow-flow phenotype. The global score seemed to separate angiographic flow phenotypes rather than mirror a continuous TFC gradient within groups, and the association was stable after adjustment for STOP-Bang screening risk. Because coronary microvascular function was not measured directly, these findings should be regarded as hypothesis-generating and do not establish a relationship between sleep quality and coronary microvascular dysfunction. They support structured assessment of sleep symptoms in patients with this phenotype and provide a clear rationale for prospective studies that integrate objective sleep metrics with invasive coronary physiology and intracoronary imaging.

## Figures and Tables

**Table 1:** Baseline clinical, laboratory, angiographic, and sleep variables according to TFC-defined flow category.

Characteristic	Normal Coronary Flow (*n*= 175)	Coronary Slow Flow (*n* = 132)	*p* Value
Age, years	54.0 [47.5–62.0]	57.0 [48.0–64.0]	0.109
Body mass index, kg/m^2^	30.3 [27.4–34.4]	29.8 [26.6–33.5]	0.206
Fasting glucose, mg/dL	99.0 [91.0–116.0]	107.5 [97.0–126.0]	0.004
HbA1c, %	5.60 [5.30–6.20]	5.70 [5.40–6.33]	0.084
Estimated GFR, mL/min/1.73 m^2^	95.0 [81.0–105.0]	94.0 [82.5–103.0]	0.623
Triglycerides, mg/dL	118.0 [88.0–159.0]	144.5 [105.0–204.2]	<0.001
LDL cholesterol, mg/dL	127.0 [100.0–150.5]	125.5 [95.2–150.0]	0.711
HDL cholesterol, mg/dL	54.0 [46.0–59.0]	44.5 [38.0–51.2]	<0.001
Albumin, g/dL	4.20 [4.00–4.47]	4.34 [4.07–4.52]	0.074
C-reactive protein, mg/dL	0.39 [0.23–0.58]	0.34 [0.20–0.52]	0.337
Hemoglobin, g/dL	13.4 [12.3–14.2]	14.1 [13.0–15.0]	<0.001
Neutrophil count, ×10^3^/µL	4.24 [3.42–5.07]	4.38 [3.47–5.16]	0.260
Platelet count, ×10^3^/µL	243.0 [198.0–278.0]	230.0 [199.8–270.2]	0.439
Lymphocyte count, ×10^3^/µL	2.26 [1.81–2.79]	2.24 [1.71–2.81]	0.380
Monocyte count, ×10^3^/µL	0.56 [0.46–0.66]	0.60 [0.45–0.74]	0.017
RDW, %	13.6 [13.0–14.4]	13.4 [12.9–14.3]	0.166
PDW, fL	12.1 [11.0–14.6]	13.0 [11.6–15.4]	0.034
MPV, fL	10.4 [9.7–11.2]	10.4 [9.8–11.2]	0.907
PCT, %	0.25 [0.21–0.30]	0.23 [0.20–0.27]	0.009
Corrected LAD TIMI frame count	22.0 [20.0–22.0]	35.0 [26.0–38.0]	<0.001
LCx TIMI frame count	19.0 [18.0–21.0]	24.0 [22.0–33.0]	<0.001
RCA TIMI frame count	21.0 [19.0–23.0]	32.0 [23.0–35.0]	<0.001
Mean TIMI frame count	20.7 [19.5–21.7]	29.0 [26.9–32.1]	<0.001
Global PSQI score	5.0 [4.0–7.0]	7.0 [6.0–8.0]	<0.001
Male sex	83 (47.4%)	86 (65.2%)	0.002
Current smoking	80 (45.7%)	92 (69.7%)	<0.001
Chronic obstructive pulmonary disease	29 (16.6%)	24 (18.2%)	0.712
Diabetes mellitus	40 (22.9%)	30 (22.7%)	0.979
Hypertension	100 (57.1%)	83 (62.9%)	0.311
Hyperlipidemia	63 (36.0%)	56 (42.4%)	0.253
Poor sleep quality, PSQI > 5	87 (49.7%)	100 (75.8%)	<0.001

Data are shown as median [interquartile range] for continuous variables and as *n* (%) for categorical variables. Group sizes (n = 175 for normal coronary flow and *n* = 132 for coronary slow flow) are given in the column headers and are complete for every variable except HbA1c. Continuous variables were analyzed with Mann–Whitney U tests, and categorical variables with Pearson chi-square or Fisher exact tests as appropriate. CSF was assigned if any vessel TFC was >27 frames after use of corrected LAD TFC, LCx TFC, and RCA TFC values; normal coronary flow required corrected LAD TFC ≤ 27 frames, LCx TFC ≤ 27 frames, and RCA TFC ≤ 27 frames. HbA1c data were available for 170 of 175 patients in the normal flow group (5 missing) and for all 132 patients in the CSF group. CSF, coronary slow flow; GFR, glomerular filtration rate; HbA1c, glycated hemoglobin; HDL, high-density lipoprotein; LAD, left anterior descending artery; LCx, left circumflex artery; LDL, low-density lipoprotein; MPV, mean platelet volume; PCT, plateletcrit; PDW, platelet distribution width; PSQI, Pittsburgh Sleep Quality Index; RCA, right coronary artery; RDW, red cell distribution width; TFC, TIMI frame count; TIMI, Thrombolysis in Myocardial Infarction.

**Table 2:** Correlations between global PSQI score and vessel-specific or mean TFC.

Variable	Overall Rho	*p*	CSF Rho	*p*	Normal Flow Rho	*p*	Partial Rho	*p*
Corrected LAD TFC	0.221	<0.001	−0.020	0.822	−0.115	0.129	−0.056	0.332
LCx TFC	0.267	<0.001	0.002	0.984	0.157	0.037	0.079	0.167
RCA TFC	0.150	0.009	−0.178	0.041	0.070	0.357	−0.067	0.242
Mean TFC	0.292	<0.001	−0.075	0.393	0.111	0.145	0.013	0.818

Partial Spearman coefficients were obtained by correlating rank residuals after adjustment for flow group membership. Vessel-level subgroup correlations that were nominally significant were small, inconsistent in direction, and not retained after flow group adjustment. CSF, coronary slow flow; LAD, left anterior descending artery; LCx, left circumflex artery; PSQI, Pittsburgh Sleep Quality Index; RCA, right coronary artery; TFC, TIMI frame count.

**Table 3:** Adjusted logistic regression model for coronary slow flow.

Variable	Adjusted OR	95% CI	*p* Value
Global PSQI score, per 1 point	1.58	1.34 to 1.88	<0.001
Age, per year	1.04	1.01 to 1.06	0.008
Male sex	1.82	1.04 to 3.19	0.036
Body mass index, per kg/m^2^	1.00	0.95 to 1.05	0.982
Current smoking	3.23	1.80 to 5.81	<0.001
Diabetes mellitus	0.96	0.51 to 1.79	0.895
Hypertension	1.55	0.89 to 2.70	0.124
Hyperlipidemia	1.40	0.80 to 2.43	0.236

The primary model included 307 participants and covariates for age, sex, body mass index, current smoking, diabetes mellitus, hypertension, and hyperlipidemia. Model diagnostics were as follows: Hosmer–Lemeshow chi-square = 20.40 (*p* = 0.009); all variance inflation factors <1.3; apparent AUC = 0.760 (bootstrap 95% CI 0.722 to 0.825). CI, confidence interval; OR, odds ratio; PSQI, Pittsburgh Sleep Quality Index.

**Table 4:** PSQI component-based models for coronary slow flow.

Domain Variable	Scale	*n*	Adjusted OR	95% CI	*p* Value	FDR q
Sleep latency > 30 min	Binary	307	2.50	1.52 to 4.11	<0.001	<0.001
Sleep duration < 6 h	Binary	307	2.89	1.69 to 4.94	<0.001	<0.001
Sleep duration < 7 h	Binary	307	4.08	2.12 to 7.86	<0.001	<0.001
Sleep efficiency < 85%	Binary	307	1.01	0.62 to 1.65	0.955	0.955
Subjective sleep quality score	Ordinal	307	1.26	0.92 to 1.71	0.145	0.266
Sleep latency score	Ordinal	307	1.93	1.46 to 2.54	<0.001	<0.001
Sleep duration score	Ordinal	307	2.16	1.59 to 2.92	<0.001	<0.001
Sleep efficiency score	Ordinal	307	0.91	0.65 to 1.27	0.593	0.805
Sleep disturbances score	Ordinal	307	0.92	0.74 to 1.15	0.454	0.713
Sleep medication score	Ordinal	307	1.11	0.70 to 1.75	0.658	0.805
Daytime dysfunction score	Ordinal	307	1.06	0.75 to 1.50	0.737	0.810

Each component model adjusted for age, sex, body mass index, current smoking, diabetes mellitus, hypertension, and hyperlipidemia. Binary domain variables and ordinal component scores were tested in separate logistic regression models; global PSQI score was intentionally omitted to avoid collinearity. FDR q values were calculated by the Benjamini–Hochberg procedure. CI, confidence interval; FDR, false discovery rate; OR, odds ratio.

## Data Availability

The data presented in this study are available on request from the corresponding author due to privacy and ethical restrictions related to patient information.

## Supplementary Materials

The following supporting information can be downloaded at: https://www.mdpi.com/article/10.3390/jcdd13080403/s1; Table S1. Available medication variables according to TFC-defined flow category; Table S2. Seven PSQI component score distributions according to TFC-defined flow category; Table S3. Sensitivity analyses for the adjusted relationship between global PSQI score and coronary slow flow; Table S4. Selected PSQI domain scores and STOP-Bang score in relation to mean TFC; Table S5. STOP-Bang total score and screening-risk category according to TFC-defined flow group; Table S6. STOP-Bang-adjusted multivariable logistic regression model for coronary slow flow; Table S7. Extended multivariable logistic regression model for coronary slow flow, additionally adjusted for all baseline variables that differed between flow groups; Table S8. Global PSQI score according to the number of vessels with corrected TIMI frame count >27; Table S9. Pharmacotherapy before coronary angiography in relation to TFC-defined flow category and to mean TIMI frame count.

## References

[1] Tambe A.A., Demany M.A., Zimmerman H.A., Mascarenhas E. (1972). Angina pectoris and slow flow velocity of dye in coronary arteries: A new angiographic finding. Am. Heart J..

[2] Beltrame J.F., Limaye S.B., Horowitz J.D. (2002). The coronary slow flow phenomenon: A new coronary microvascular disorder. Cardiology.

[3] Wang X., Nie S.P. (2011). The coronary slow flow phenomenon: Characteristics, mechanisms and implications. Cardiovasc. Diagn. Ther..

[4] Hawkins B.M., Stavrakis S., Rousan T.A., Abu Fadel M., Schechter E. (2012). Coronary slow flow: Prevalence and clinical correlations. Circ. J..

[5] Chalikias G., Tziakas D. (2021). Slow coronary flow: Pathophysiology, clinical implications, and therapeutic management. Angiology.

[6] Zhu Q., Wang S., Huang X., Zhao C., Wang Y., Li X., Jia D., Ma C. (2024). Understanding the pathogenesis of coronary slow flow: Recent advances. Trends Cardiovasc. Med..

[7] Wang D., Li Y., Hu J., Zhang K. (2026). Coronary slow flow phenomenon: A meta-analysis of clinical risk predictors. Front. Cardiovasc. Med..

[8] Gibson C.M., Cannon C.P., Daley W.L., Dodge J.T., Alexander B., Marble S.J., McCabe C.H., Raymond L., Fortin T., Poole W.K. (1996). TIMI frame count: A quantitative method of assessing coronary artery flow. Circulation.

[9] Buysse D.J., Reynolds C.F., Monk T.H., Berman S.R., Kupfer D.J. (1989). The Pittsburgh Sleep Quality Index: A new instrument for psychiatric practice and research. Psychiatry Res..

[10] Agargun M.Y., Kara H., Anlar O. (1996). The validity and reliability of the Pittsburgh Sleep Quality Index. Turk. Psikiyatr. Derg..

[11] Lloyd-Jones D.M., Allen N.B., Anderson C.A.M., Black T., Brewer L.C., Foraker R.E., Grandner M.A., Lavretsky H., Perak A.M., Sharma G. (2022). Life’s Essential 8: Updating and enhancing the American Heart Association’s construct of cardiovascular health. Circulation.

[12] Watson N.F., Badr M.S., Belenky G., Bliwise D.L., Buxton O.M., Buysse D., Dinges D.F., Gangwisch J., Grandner M.A., Kushida C. (2015). Recommended amount of sleep for a healthy adult: A joint consensus statement of the American Academy of Sleep Medicine and Sleep Research Society. Sleep.

[13] Holmer B.J., Lapierre S.S., Jake-Schoffman D.E., Christou D.D. (2021). Effects of sleep deprivation on endothelial function in adult humans: A systematic review. GeroScience.

[14] Jaspan V.N., Greenberg G.S., Parihar S., Park C.M., Somers V.K., Shapiro M.D., Lavie C.J., Virani S.S., Slipczuk L. (2024). The role of sleep in cardiovascular disease. Curr. Atheroscler. Rep..

[15] Ozeke O., Gungor M., Ertan C., Celik A., Aydin D., Erturk O., Hizel S.B., Ozgen F., Demir A.D., Ozer C. (2012). Association of sleep apnea with coronary slow-flow phenomenon. J. Cardiovasc. Med..

[16] Ocal A., Saricam E., Basay N., Sariyildiz G. (2020). The coexistence of obstructive sleep apnea in patients with slow coronary flow: A cross-sectional study. Anatol. Curr. Med. J..

[17] Durmaz T., Keles T., Erdogan K.E., Ayhan H., Bilen E., Bayram N.A., Akcay M., Oz O., Albayrak Y., Ozdemir N. (2014). Coronary slow flow is associated with depression and anxiety. Acta Cardiol. Sin..

[18] Elamragy A.A., Abdelhalim A.A., Arafa M.E., Baghdady Y.M. (2019). Anxiety and depression relationship with coronary slow flow. PLoS ONE.

[19] Ozturk H.M., Inanc I.H., Cilingiroglu M., Turan Y., Kandemir H., Ozturk S. (2025). Coronary slow flow is associated with anxiety and depression but not adverse childhood experiences and alexithymia. J. Mind Med. Sci..

[20] Lao X.Q., Liu X., Deng H.B., Chan T.C., Ho K.F., Wang F., Vermeulen R., Tam T., Wong M.C., Tse L. (2018). Sleep quality, sleep duration, and the risk of coronary heart disease: A prospective cohort study with 60,586 adults. J. Clin. Sleep Med..

[21] Yang J., Wang K., Wang W., Niu J., Liu X., Shen H., Sun Y., Ge H., Han H. (2024). The effect of sleep quality on coronary lesion severity and prognosis in the young acute coronary syndrome population. J. Cardiovasc. Dev. Dis..

[22] Yarlioglues M., Karacali K., Ilhan B.C., Yalcinkaya Oner D. (2024). An observational study: The relationship between sleep quality and angiographic progression in patients with chronic coronary artery disease. Sleep Med..

[23] Von Kanel R., Princip M., Holzgang S.A., Rossi A., Giannopoulos A.A., Buechel R.R., Zuccarella-Hackl C., Pazhenkottil A.P. (2024). Association between global sleep quality and coronary microvascular function in male physicians with occupational burnout. Psychosom. Med..

[24] Chung F., Yegneswaran B., Liao P., Chung S.A., Vairavanathan S., Islam S., Khajehdehi A., Shapiro C.M. (2008). STOP questionnaire: A tool to screen patients for obstructive sleep apnea. Anesthesiology.

[25] Chung F., Subramanyam R., Liao P., Sasaki E., Shapiro C., Sun Y. (2012). High STOP-Bang score indicates a high probability of obstructive sleep apnoea. Br. J. Anaesth..

[26] Pivetta B., Chen L., Nagappa M., Saripella A., Waseem R., Englesakis M., Chung F. (2021). Use and performance of the STOP-Bang questionnaire for obstructive sleep apnea screening across geographic regions: A systematic review and meta-analysis. JAMA Netw. Open.

[27] Yeghiazarians Y., Jneid H., Tietjens J.R., Redline S., Brown D.L., El-Sherif N., Mehra R., Bozkurt B., Ndumele C.E., Somers V.K. (2021). Obstructive sleep apnea and cardiovascular disease: A scientific statement from the American Heart Association. Circulation.

[28] Faul F., Erdfelder E., Buchner A., Lang A.G. (2009). Statistical power analyses using G*Power 3.1: Tests for correlation and regression analyses. Behav. Res. Methods.

[29] Demidenko E. (2007). Sample size determination for logistic regression revisited. Stat. Med..

[30] Peduzzi P., Concato J., Kemper E., Holford T.R., Feinstein A.R. (1996). A simulation study of the number of events per variable in logistic regression analysis. J. Clin. Epidemiol..

[31] Benjamini Y., Hochberg Y. (1995). Controlling the false discovery rate: A practical and powerful approach to multiple testing. J. R Stat. Soc. Ser. B Stat. Methodol..

[32] von Elm E., Altman D.G., Egger M., Pocock S.J., Gotzsche P.C., Vandenbroucke J.P. (2007). The Strengthening the Reporting of Observational Studies in Epidemiology statement: Guidelines for reporting observational studies. Lancet.

[33] Huang Q., Zhang F., Chen S., Dong Z., Liu W., Zhou X. (2021). Clinical characteristics in patients with coronary slow flow phenomenon: A retrospective study. Medicine.

[34] Tu G., Zhao C., Cai Z.L., Huang X.M., Tong S.Y., Wang N., Qian J.M. (2024). Development and validation of a nomogram diagnostic model for coronary slow flow patients: A cross-sectional study. Medicine.

[35] Farsi F., Morovatdar N., Eshraghi A. (2025). Baseline characteristics and seven-year follow-up of patients with coronary slow flow: A cohort study in northeastern Iran. J. Cardiovasc. Thorac. Res..

[36] Edinger J.D., Arnedt J.T., Bertisch S.M., Carney C.E., Harrington J.J., Lichstein K.L., Sateia M.M.J., Troxel W.M., Zhou E.S., Kazmi U. (2021). Behavioral and psychological treatments for chronic insomnia disorder in adults: An American Academy of Sleep Medicine clinical practice guideline. J. Clin. Sleep Med..

[37] Mayer M., Allan T., Harkin K.L., Loftspring E., Saffari S.E., Reynolds H.R., Paul J., Kalathiya R., Shah A.P., Nathan S. (2024). Angiographic coronary slow flow is not a valid surrogate for invasively diagnosed coronary microvascular dysfunction. JACC Cardiovasc. Interv..

