# Supplementary material for: Association Between Pittsburgh Sleep Quality Index Scores and TIMI Frame Count-Defined Coronary Slow Flow Phenomenon in Patients with Nonobstructive Coronary Arteries: A Cross-Sectional Study
Source: J Cardiovasc Dev Dis. 2026 Aug 21;13(8):403. doi: 10.3390/jcdd13080403 (PMC13513871; doi:10.3390/jcdd13080403)
Supplement: Supplementary file 1 [file jcdd-13-00403-s001.zip › jcdd-4450356-supplementary.pdf]

Supplementary Materials

Table S1. Available medication variables according to TFC-defined flow category

Variable	Normal coronary flow	Coronary slow flow	P value
ACE inhibitor or ARB	92/175 (52.6%)	72/132 (54.5%)	0.731
Beta blocker	75/175 (42.9%)	65/132 (49.2%)	0.266
Calcium channel blocker	56/175 (32.0%)	38/132 (28.8%)	0.545
Diuretic	56/175 (32.0%)	38/132 (28.8%)	0.545
Statin	63/174 (36.2%)	58/132 (43.9%)	0.171
Aspirin	93/175 (53.1%)	61/132 (46.2%)	0.229
Additional antiplatelet	11/175 (6.3%)	4/132 (3.0%)	0.190
Oral anticoagulant	10/175 (5.7%)	18/132 (13.6%)	0.017
Antidiabetic therapy	40/175 (22.9%)	31/132 (23.5%)	0.897

Data are presented as n/N (%). Pearson chi-square or Fisher exact tests were used as appropriate. Medication variables were described for context and entered into sensitivity analyses rather than the primary model. ACE, angiotensin-converting enzyme; ARB, angiotensin receptor blocker; CSF, coronary slow flow.

Table S2. Seven PSQI component score distributions according to TFC-defined flow category

PSQI component	Normal median [IQR]	Normal score 0/1/2/3	CSF median [IQR]	CSF score 0/1/2/3	P value
C1 subjective sleep quality	0.0 [0.0-1.0]	90/60/22/3	1.0 [0.0-1.0]	55/56/14/7	0.115
C2 sleep latency	1.0 [0.0-2.0]	54/62/48/11	2.0 [1.0-2.0]	14/42/46/30	<0.001
C3 sleep duration	1.0 [0.0-1.0]	59/75/33/8	1.0 [1.0-2.0]	16/56/39/21	<0.001
C4 habitual sleep efficiency	0.0 [0.0-1.0]	95/51/29/0	0.0 [0.0-1.0]	68/47/17/0	0.918
C5 sleep disturbances	1.0 [0.0-2.0]	45/54/36/40	1.0 [0.0-2.0]	44/23/44/21	0.501
C6 sleep medication	0.0 [0.0-0.0]	135/32/8/0	0.0 [0.0-1.0]	98/29/5/0	0.603
C7 daytime dysfunction	0.0 [0.0-1.0]	98/56/21/0	0.0 [0.0-1.0]	67/48/17/0	0.403

Component scores range from 0 to 3, and higher scores indicate greater impairment in the relevant domain. Ordinal score distributions were compared with Mann–Whitney U tests. CSF, coronary slow flow; IQR, interquartile range; PSQI, Pittsburgh Sleep Quality Index.

Table S3. Sensitivity analyses for the adjusted relationship between global PSQI score and coronary slow flow

Model	n	PSQI adjusted OR	95% CI	P value	Model AUC

Main clinical model	307	1.58	1.34 to 1.88	<0.001	0.760
STOP-Bang total added	307	1.62	1.36 to 1.93	<0.001	0.760
STOP-Bang ≥3 risk category added	307	1.60	1.35 to 1.90	<0.001	0.759
Without body mass index	307	1.58	1.34 to 1.87	<0.001	0.760
Without hyperlipidemia	307	1.59	1.34 to 1.88	<0.001	0.761
With triglycerides and HDL	307	1.56	1.31 to 1.86	<0.001	0.803
With statin and oral anticoagulant	306	1.58	1.33 to 1.87	<0.001	0.769

Each model contained global PSQI score and the covariates indicated by the model label. The medication-adjusted model included statin and oral anticoagulant variables. AUC, area under the receiver operating characteristic curve; CI, confidence interval; OR, odds ratio; PSQI, Pittsburgh Sleep Quality Index.

Table S4. Selected PSQI domain scores and STOP-Bang score in relation to mean TFC

Domain	Overall rho	Overall P	CSF rho	CSF P	Normal flow rho	Normal flow P	Partial rho	Partial P
Sleep latency score	0.243	<0.001	-0.071	0.420	0.021	0.782	-0.024	0.677
Sleep duration score	0.241	<0.001	-0.031	0.726	0.010	0.899	-0.016	0.778
Sleep efficiency score	-0.007	0.900	-0.077	0.383	0.010	0.895	-0.022	0.696
STOP-Bang total score	0.079	0.169	-0.079	0.366	0.098	0.199	0.024	0.675

Partial Spearman correlations were calculated from rank residuals after adjustment for flow group membership. CSF, coronary slow flow; PSQI, Pittsburgh Sleep Quality Index; TFC, TIMI frame count.

Table S5. STOP-Bang total score and screening-risk category according to TFC-defined flow group

Variable	Normal flow n	Normal coronary flow	CSF n	Coronary slow flow	P value

STOP-Bang total score	175	3.0 [2.0-4.0]	132	3.0 [2.0-4.0]	0.172
STOP-Bang ≥3 risk category	175	101/175 (57.7%)	132	87/132 (65.9%)	0.145

STOP-Bang total score was used as a screening estimate of obstructive sleep apnea risk. STOP-Bang ≥3 was described as an elevated screening-risk category. Continuous variables were compared with Mann–Whitney U tests, and categorical variables with Pearson chi-square or Fisher exact tests.

Table S6. STOP-Bang-adjusted multivariable logistic regression model for coronary slow flow

Variable	Adjusted OR	95% CI	P value
Global PSQI score, per 1 point	1.62	1.36 to 1.93	<0.001
Age, per year	1.04	1.01 to 1.07	0.003
Male sex	2.48	1.24 to 4.93	0.010
Body mass index, per kg/m²	1.02	0.96 to 1.08	0.478
Current smoking	3.15	1.75 to 5.67	<0.001
Diabetes mellitus	1.00	0.54 to 1.88	0.990
Hypertension	1.92	1.03 to 3.60	0.040
Hyperlipidemia	1.41	0.81 to 2.46	0.227
STOP-Bang total score, per 1 point	0.81	0.62 to 1.06	0.124

The model contained global PSQI score, age, sex, body mass index, current smoking, diabetes mellitus, hypertension, hyperlipidemia, and STOP-Bang total score. CI, confidence interval; OR, odds ratio; PSQI, Pittsburgh Sleep Quality Index.

Table S7. Extended multivariable logistic regression model for coronary slow flow, additionally adjusted for all baseline variables that differed between flow groups

Variable	Adjusted OR	95% CI	P value
Global PSQI score, per 1 point	1.59	1.33 to 1.90	<0.001
Age, per 1 SD (10.6 years)	1.48	1.10 to 2.00	0.010
Male sex	0.82	0.40 to 1.67	0.577
Body mass index, per 1 SD (5.1 kg/m²)	0.97	0.73 to 1.29	0.836
Current smoking	2.95	1.56 to 5.55	<0.001
Diabetes mellitus	0.39	0.15 to 0.99	0.048
Hypertension	1.41	0.77 to 2.59	0.265
Hyperlipidemia	1.69	0.92 to 3.07	0.089
Fasting glucose, per 1 SD (48.9 mg/dL)	1.31	0.89 to 1.91	0.172
Triglycerides, per 1 SD (92.6 mg/dL)	1.54	1.09 to 2.18	0.014
HDL cholesterol, per 1 SD (13.3 mg/dL)	0.68	0.49 to 0.95	0.024
Hemoglobin, per 1 SD (1.51 g/dL)	1.27	0.89 to 1.80	0.187

Monocyte count, per 1 SD (0.17 $\times 10^3/\mu L$)	1.35	1.00 to 1.82	0.047
Platelet distribution width, per 1 SD (2.35 fL)	1.28	0.96 to 1.70	0.090
Plateletcrit, per 1 SD (0.062%)	0.71	0.52 to 0.97	0.033

The model contained the covariates of the primary model together with every baseline variable that differed significantly between flow groups in Table 1. Continuous laboratory variables are expressed per 1 standard deviation to avoid odds ratios referring to implausible one-unit changes. Model characteristics: n = 307; 132 events for 15 covariates (8.8 events per variable); apparent AUC = 0.829; Hosmer–Lemeshow chi-square = 5.56, P = 0.697; maximum variance inflation factor = 1.95. The inverse association observed for diabetes mellitus should be interpreted with caution, because fasting glucose was included in the same model and the two variables are closely related. CI, confidence interval; OR, odds ratio; PSQI, Pittsburgh Sleep Quality Index.

Table S8. Global PSQI score according to the number of vessels with corrected TIMI frame count >27

Number of vessels with TFC >27	n	Global PSQI score, median [IQR]
0 (normal coronary flow)	175	5.0 [4.0-7.0]
1	67	7.0 [6.0-8.0]
2	41	7.0 [6.0-8.0]
3	24	5.5 [4.0-7.0]

Across all four categories, Kruskal–Wallis P < 0.001, driven by the difference between patients with no affected vessel and those with any affected vessel. Restricted to the 132 patients with CSF, global PSQI score did not increase with the number of affected vessels (Kruskal–Wallis P = 0.082; Spearman rho = -0.134, P = 0.125). IQR, interquartile range; PSQI, Pittsburgh Sleep Quality Index; TFC, TIMI frame count.

Table S9. Pharmacotherapy before coronary angiography in relation to TFC-defined flow category and to mean TIMI frame count

Medication	Normal coronary flow	Coronary slow flow	P value	Adjusted OR for CSF (95% CI)	P value	Mean TFC, users	Mean TFC, non-users	P value
ACE inhibitor or ARB	92/175 (52.6%)	72/132 (54.5%)	0.731	0.96 (0.40 to 2.31)	0.920	22.7 [20.3-28.7]	22.0 [20.3-28.0]	0.689
Beta blocker	75/175 (42.9%)	65/132 (49.2%)	0.266	1.09 (0.63 to 1.88)	0.768	22.7 [20.7-28.4]	22.0 [20.0-28.0]	0.599
Calcium channel blocker	56/175 (32.0%)	38/132 (28.8%)	0.545	0.81 (0.42 to 1.55)	0.528	22.0 [20.3-28.4]	22.7 [20.3-28.0]	0.797
Diuretic	56/175 (32.0%)	38/132 (28.8%)	0.545	0.93 (0.48 to 1.80)	0.820	22.5 [21.0-28.7]	22.0 [20.0-28.0]	0.351

Statin	63/174 (36.2%)	58/132 (43.9%)	0.171	1.01 (0.31 to 3.34)	0.982	23.3 [20.0-29.3]	22.0 [20.3-27.7]	0.509
Aspirin	93/175 (53.1%)	61/132 (46.2%)	0.229	0.57 (0.33 to 1.00)	0.050	22.0 [20.1-27.3]	22.7 [20.3-28.3]	0.235
Additional antiplatelet	11/175 (6.3%)	4/132 (3.0%)	0.190	0.35 (0.08 to 1.48)	0.153	20.3 [19.3-25.5]	22.7 [20.3-28.0]	0.063
Oral anticoagulant	10/175 (5.7%)	18/132 (13.6%)	0.017	2.52 (1.00 to 6.39)	0.051	26.7 [21.9-30.6]	22.0 [20.3-27.8]	0.007
Antidiabetic therapy	40/175 (22.9%)	31/132 (23.5%)	0.897	0.96 (0.52 to 1.80)	0.905	22.7 [20.3-27.5]	22.2 [20.3-28.3]	0.847

Categorical data are n/N (%) and were compared with Pearson chi-square or Fisher exact tests as appropriate. Adjusted odds ratios were obtained by adding each medication variable individually to the primary clinical model, which contained global PSQI score, age, sex, body mass index, current smoking, diabetes mellitus, hypertension, and hyperlipidemia. Because antidiabetic therapy and the diabetes covariate were discordant in only 1 of 307 patients, antidiabetic therapy was entered in place of the diabetes covariate rather than alongside it. Mean TFC values are median [interquartile range] frames and were compared with Mann–Whitney U tests. Statin data were missing for 1 patient in the normal-flow group; the reported denominator reflects available data. When all medication variables were entered into the primary model simultaneously, the adjusted odds ratio for global PSQI score was 1.57 (95% confidence interval 1.32 to 1.87, $P < 0.001$). ACE, angiotensin-converting enzyme; ARB, angiotensin receptor blocker; CI, confidence interval; CSF, coronary slow flow; OR, odds ratio; PSQI, Pittsburgh Sleep Quality Index; TFC, TIMI frame count.
